# Health on the web: a randomised controlled trial of work-based online screening and brief intervention for reducing alcohol intake

**DOI:** 10.1186/1940-0640-8-S1-A40

**Published:** 2013-09-04

**Authors:** Zarnie Khadjesari, Elizabeth Murray, Stuart Linke, Rachael Hunter, Nick Freemantle

**Affiliations:** 1 Research Department of Primary Care and Population Health, University College London, London, UK; 2 Camden and Islington NHS Foundation Trust, London, UK

## Introduction

An estimated 11-17 million work days are lost annually in Britain due to alcohol-related sickness, with total costs to the workplace of £6.4bn (compared with £1.7bn to health services). The workplace provides an ideal setting for online screening and brief intervention, with access to a potentially large sample of adults from varying socioeconomic groups. The aim of this study was to determine the effectiveness of online screening and brief intervention for alcohol misuse within a workplace setting.

## Methods

Employees of a large company were invited to take part in an online health check for a range of behaviours, including smoking, diet, physical activity and alcohol consumption. No further baseline data were collected to minimise reactivity of assessment. Employees drinking above recommended limits (≥5 AUDIT-C) were entered into the trial. The intervention group received feedback on all behaviours, including recommendation of Down Your Drink (DYD) (an extensive online intervention). The control group received feedback on all behaviours except alcohol intake. The primary outcome was past week alcohol consumption. Secondary outcomes included AUDIT, EQ-5D, days off work, number and duration of hospital admissions and use of DYD.

## Results

3,375 employees took part in the health check, of which 1,330 (39%) scored ≥5 AUDIT-C. Follow-up data were collected for 80% of participants at 3 months. Participants were mostly male (75%), married (77%) with relatively healthy lifestyles. There was no significant difference between groups for any outcome. Both groups reduced their median AUDIT-C score by 1. Only 19 participants registered with the DYD website for further help to reduce their drinking.

## Conclusions

In the context of an online health check, this pragmatic trial did not find brief advice to reduce alcohol intake when compared with no feedback. Completion of the AUDIT-C alone may have led both groups to reduce their drinking slightly.

